# Sexual disfunction in adolescents with antipsychotics. evaluation and suitability of the treatment

**DOI:** 10.1192/j.eurpsy.2021.1376

**Published:** 2021-08-13

**Authors:** S. Pérez Sánchez, I. Martín Herrero, M.A. Cutillas Fernández, D. Raya Güimil, A. Crespo Portero

**Affiliations:** Servicio De Psiquiatría, Servicio Murciano de Salud, Murcia, Spain

**Keywords:** ADOLESCENTS, ANTIPSYCHOTICS, SEXUAL DYSFUNCTION, ARIPIPRAZOLE

## Abstract

**Introduction:**

Problems in sexual function associated with psychotropic drugs in adolescents with psychotic disorders are common in clinical practice. However, they are usually not taken into account in follow-up and are rarely reported by patients.

**Objectives:**

1. To analyze if there is sexual dysfunction in adolencents with antipsychotic treatment. 2. To assess the degree of sexual dysfunction.

**Methods:**

Descriptive study in psychiatric outpatient clinics involving 14 men (aged 16 to 19) with antipsychotic treatment. Record prospectively through interviews between 2 and 4 months from the start of treatment. Sexual function was evaluated with the questionnaire SALSEX Informed consent.

**Results:**

Initially, no sexual dysfunction scores are obtained. At 4 months, records of sexual dysfunction were observed in 67% of the patients with less impact in those with aripiprazole as antipsychotic treatment, with a moderate intensity (mean score 8.2; SD 4.7). 33% of cases report the problem spontaneously. Breaking down the reasons for sexual dysfunction: 50% decrease in libido, 20% delay in ejaculation and 7% impotence. The global tolerance to sexual dysfunction was poor, 45% with ideas to abandon treatment.

**Conclusions:**

In our experience, sexual dysfunction is one of the main causes that make young patients abandon treatment and even follow-up. For what we consider, it is very relevant to systematically evaluate and be able to quantify this vital aspect of our patients, which on many occasions is not addressed in the consultation. Likewise, it will be necessary in future studies to describe in detail the psychotropic drugs associated with sexual dysfunction for better management and dose adjustment.

